# Origin of the emergence of higher *T*_c_ than bulk in iron chalcogenide thin films

**DOI:** 10.1038/s41598-017-10383-1

**Published:** 2017-08-30

**Authors:** Sehun Seo, Jong-Hoon Kang, Myeong Jun Oh, Il-Seok Jeong, Jianyi Jiang, Genda Gu, Jung-Woo Lee, Jongmin Lee, Heesung Noh, Mengchao Liu, Peng Gao, Eric E. Hellstrom, Joo-Hyoung Lee, Youn Jung Jo, Chang-Beom Eom, Sanghan Lee

**Affiliations:** 1 School of Materials Science and Engineering, Gwangju Institute of Science and Technology, Gwangju, 61005 South Korea; 2 Department of Materials Science and Engineering, University of Wisconsin-Madison, Madison, Wisconsin 53706 USA; 3 Department of Physics, Kyungpook National University, Daegu, 41566 South Korea; 4 Applied Superconductivity Center, National High Magnetic Field Laboratory, Florida State University, Tallahassee, Florida 32310 USA; 5 Condensed Matter Physics and Materials Science Department, Brookhaven National Laboratory, Upton, New York, 11973 USA; 6 Electron Microscopy Laboratory, School of Physics, Peking University, Beijing, 100871 China

## Abstract

Fabrication of epitaxial FeSe_x_Te_1−x_ thin films using pulsed laser deposition (PLD) enables improving their superconducting transition temperature (*T*_c_) by more than ~40% than their bulk *T*_c_. Intriguingly, *T*_c_ enhancement in FeSe_x_Te_1−x_ thin films has been observed on various substrates and with different Se content, *x*. To date, various mechanisms for *T*_c_ enhancement have been reported, but they remain controversial in universally explaining the *T*_c_ improvement in the FeSe_x_Te_1−x_ films. In this report, we demonstrate that the controversies over the mechanism of *T*_c_ enhancement are due to the abnormal changes in the chalcogen ratio (Se:Te) during the film growth and that the previously reported *T*_c_ enhancement in FeSe_0.5_Te_0.5_ thin films is caused by a remarkable increase of Se content. Although our FeSe_x_Te_1−x_ thin films were fabricated via PLD using a Fe_0.94_Se_0.45_Te_0.55_ target, the precisely measured composition indicates a Se-rich FeSe_x_Te_1−x_ (0.6 < *x < *0.8) as ascertained through accurate compositional analysis by both wavelength dispersive spectroscopy (WDS) and Rutherford backscattering spectrometry (RBS). We suggest that the origin of the abnormal composition change is the difference in the thermodynamic properties of ternary FeSe_x_Te_1−x_, based on first principle calculations.

## Introduction

Iron chalcogenide superconductors (FeSe_x_Te_1−x_) have attracted considerable interest due to their enhanced superconducting critical transition temperatures (*T*_c_) in epitaxial FeSe_x_Te_1−x_ thin films fabricated by pulsed laser deposition (PLD) as compared to bulk FeSe_x_Te_1−x_^1–10^. In the early stages, since bulk FeSe_x_Te_1−x_ (x = 0.5) has a maximum *T*_c_ of about 15 K^2, 11^, the growth and characterization of FeSe_0.5_Te_0.5_ thin films were pursued vigorously. Interestingly, enhanced *T*_c_ values of 18–21 K have been consistently observed in most of the FeSe_0.5_Te_0.5_ thin films^3–6, 12^. In addition, this improved *T*_c_ has been observed in FeSe_x_Te_1−x_ thin films with Se content *x* different from 0.5^9, 10, 13^. Thus, fabricating FeSe_x_Te_1−x_ thin films using PLD is advantageous for practical applications, owing to their enhanced *T*_c_. However, the origin and mechanism of the *T*_c_ enhancement in FeSe_x_Te_1−x_ thin films remain controversial.

One contentious issue is why the *T*_c_s of FeSe_x_Te_1−x_ thin films are considerably enhanced by over 40% than bulk *T*_c_, regardless of the substrate and their composition. So far, diverse mechanisms have been proposed depending on the substrates and compositions used. In case of FeSe_0.5_Te_0.5_ thin films on LaAlO_3_ substrate, the origin of the enhanced *T*_c_ was suggested to be the biaxial strain caused by the Volmer-Weber growth mode^4^. In FeSe_0.5_Te_0.5_ thin films on CaF_2_, the *T*_c_ enhancement was attributed to the lattice contraction along all axes via the substitution of Se^2−^ ions by F^−^ ions at the interface^8, 9, 14^. Lately, several researchers have suggested that the suppression of phase separation causes improved *T*_c_ of Se rich FeSe_x_Te_1−x_ thin films (0.6 ≤ x ≤ 0.8) on CaF_2_ substrate using superconducting phase diagrams of FeSe_x_Te_1−x_ thin films. These phase diagrams have been obtained by fabricating FeSe_x_Te_1−x_ thin films on CaF_2_ using FeSe_x_Te_1−x_ targets with different Se contents, and they indicate that *T*_c_ increases with increasing Se content *x*, until *x* = 0.8^9, 10^. In spite of diverse mechanisms, each fails to explain how *T*_c_ of FeSe_x_Te_1−x_ thin films is enhanced significantly than the bulk *T*_c_.

Interestingly, most of the lattice constants previously reported for FeSe_0.5_Te_0.5_ thin films with enhanced *T*_c_ were found to be reduced along all axes compared to those of bulk FeSe_0.5_Te_0.5_, and these lattice constants are similar to the reported Se rich FeSe_x_Te_1−x_ thin films (0.6 ≤ x ≤ 0.8). We presume that the controversies with respect to the origin of the *T*_c_ enhancement in FeSe_x_Te_1−x_ thin films are due to the compositional changes during their fabrication by PLD. To date, the composition of FeSe_x_Te_1−x_ thin films has been considered to be similar to that of the bulk targets used because not only is PLD considered an effective method for fabricating stoichiometric thin films, but also the measurement of the accurate composition of the fabricated films using a general method such as scanning electron microscopy with energy dispersive X-ray spectroscopy (SEM/EDS) is significantly difficult^9^. However, compositional variations in FeSe_x_Te_1−x_ thin films is reasonably possible as a result of the sensitivity of chalcogen and its compounds, considering the thermodynamic properties such as formation energy, melting point, and vapour pressure^6, 15–17^. In addition, if the composition of FeSe_x_Te_1−x_ is changed during the thin film growth, superconducting properties can be affected because the chalcogen ratio (Se:Te) and supersaturation of Fe significantly affect the superconducting properties of both FeSe_x_Te_1−x_ bulk and thin films^2, 9, 10, 18, 19^. Furthermore, changes in the chalcogen ratio (Se:Te) causes changes in the lattice constants of the material owing to the different atomic sizes of Se and Te. Thus, measuring the accurate composition of FeSe_x_Te_1−x_ thin films is crucial to understand the mechanism underlying superconductivity enhancement and to resolve controversies regarding the origin of the lattice contraction.

In this paper, we report that the enhanced *T*_c_ and lattice contraction along all axes are caused by a remarkable increase in Se content *x* in FeSe_x_Te_1−x_ thin films. To confirm the lattice contraction along all the axes, we fabricated epitaxial FeSe_x_Te_1−x_ thin films on CaF_2_ substrate by PLD using a Fe_0.94_Se_0.45_Te_0.55_ target, and lattice contraction was confirmed by high-resolution X-ray diffraction (HR-XRD) and reciprocal space mapping (RSM). EDS is widely used to determine material compositions. However, accurate measurement of the composition presents significant difficulties such as low spectral resolution and peak overlap between the film and substrate. Therefore, we used wavelength dispersive spectroscopy (WDS) to accurately measure compositions of the films, and one of the WDS results was verified by Rutherford backscattering spectrometry (RBS) because of its higher spectral resolution and quantification accuracy. The measured composition of our FeSe_x_Te_1−x_ thin films indicates a large increase in Se content (*x*) up to ~0.7, although these films were fabricated using the Fe_0.94_Se_0.45_Te_0.55_ target. The fabricated Se-rich FeSe_x_Te_1−x_ thin films (0.6 < x < 0.8) show enhanced *T*_c_ than bulk samples, and the maximum onset superconducting transition temperature (*T*_c,onset_) of our samples is as high as 22 K. We believe that the remarkable increase in the Se content is closely related to the thermodynamic properties of FeSe_x_Te_1−x_. Moreover, we also demonstrate a mutual relationship between the chalcogen ratio and superconducting properties.

## Results and Discussion

**Figure 1:**
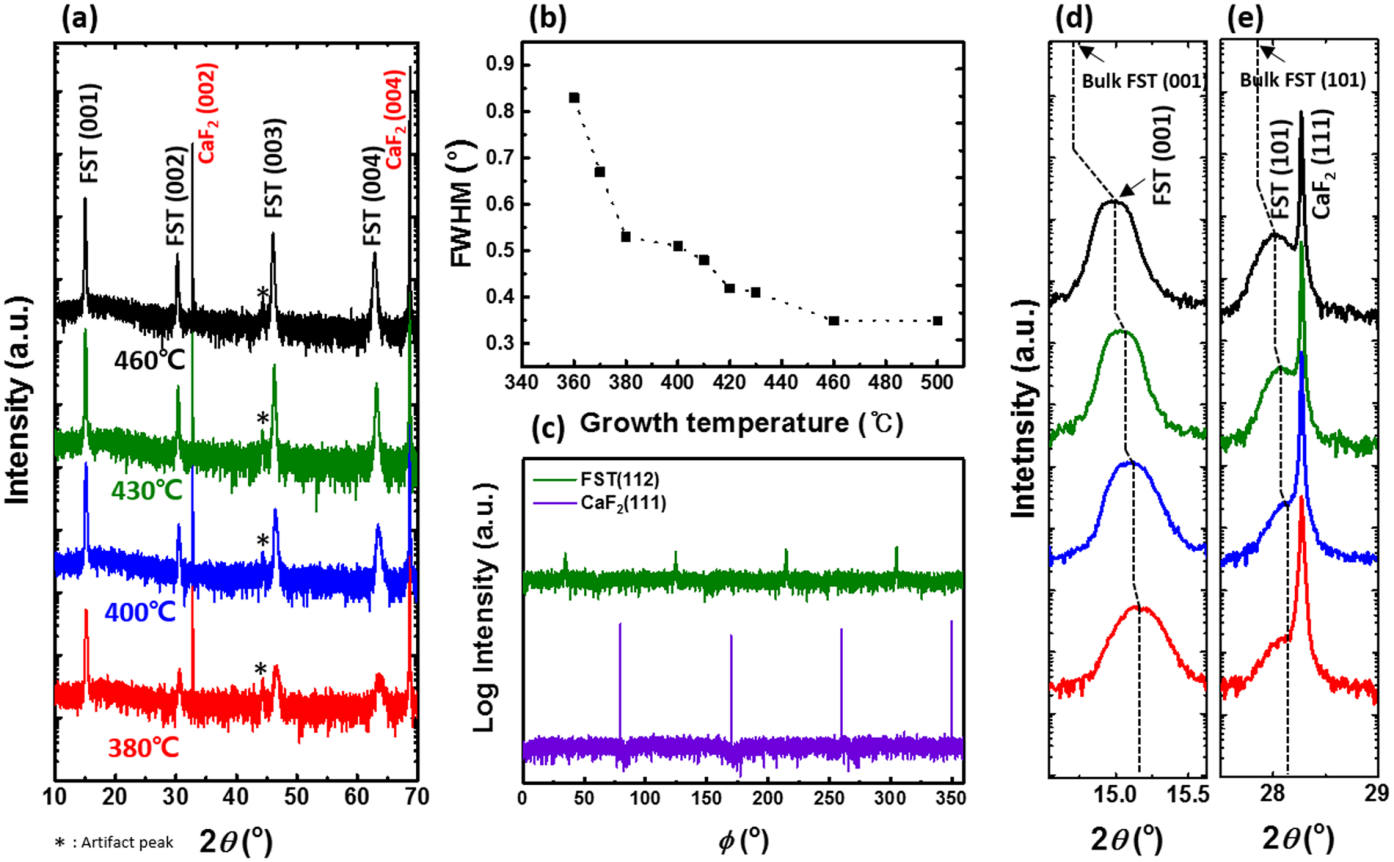
Structural analysis of FeSe_x_Te_1−x_ thin films. (**a**) Out-of-plane *θ–*2*θ* scan of the FeSe_x_Te_1−x_ (FST) thin films grown at various temperature and (**b**) FWHM of the rocking curve on (001) reflection from FeSe_x_Te_1−x_ thin films. (**c**) Azimuthal *ϕ* scan of the off-axis (112) reflection from FeSe_x_Te_1−x_ thin films grown at 430 °C. For peak shift verification, (**d**) FeSe_x_Te_1−x_ (001) and (**e**) FeSe_x_Te_1−x_ (101) reflections are magnified. Dashed line indicates the peak positions of FeSe_x_Te_1−x_ bulk and thin films and the peak shift is marked in detail. The “artifact peak” means that the peak is originated by substrate holder glue in our XRD system.

In order to investigate the lattice contraction along all axes in FeSe_x_Te_1−x_ thin films, we have fabricated FeSe_x_Te_1−x_ films on CaF_2_ substrate at various growth temperatures, using a Fe_0.94_Se_0.45_Te_0.55_ target, and have analysed the structure and crystalline quality of the FeSe_x_Te_1−x_ films by four-circle X-ray diffraction (XRD) analysis [Fig. 1]. Figure 1(a) shows out-of-plane *θ–*2*θ* scan of FeSe_x_Te_1−x_ thin films. Only FeSe_x_Te_1−x_ (00 *l*) reflections are observed along with the CaF_2_ substrate (00 *l*) reflections in all samples, indicating that the FeSe_x_Te_1−x_ thin films are well oriented along the *c*-axis. However, full width at half maximum (FWHM) of the FeSe_x_Te_1−x_ (001) rocking curve, which determines the crystalline quality and mosaic spread, is broader about 0.3 to 0.9 with decreasing growth temperature, as shown in Fig. 1(b). To verify the presence of secondary phases and to study the in-plane FeSe_x_Te_1−x_ structure in detail, additional *θ–*2*θ* scans were carried out using a two-dimensional XRD system and XRD in the acceleration laboratory. When an additional *θ–*2*θ* scan of a FeSe_x_Te_1−x_ film grown at 380 °C was carried out, only (00 *l*) reflections were observed without any in-plane structure or phase separation, indicating that the out-of-plane epitaxial arrangements are good (see Supplementary Fig. S1). Furthermore, the in-plane epitaxial arrangement and crystalline quality of FeSe_x_Te_1−x_ thin films were determined by the azimuthal *ϕ* scan of the off-axis FeSe_x_Te_1−x_ (112) reflection of the FeSe_x_Te_1−x_ thin film grown at 430 °C, as shown in Fig. 1(c). Since strong and sharp peaks appear at intervals of 90° without any extra peaks, it is confirmed that the FeSe_x_Te_1−x_ thin films have a good epitaxial arrangement in in-plane orientation without any misoriented grains. Thus, the fabricated FeSe_x_Te_1−x_ samples are high quality single-crystal-like materials without any secondary phases or phase separation. Figure 1(d) shows an enlarged section of Fig. 1(a) close to the (001) reflection of the FeSe_x_Te_1−x_ films. Interestingly, FeSe_x_Te_1−x_ (00 l) reflections are noticeably shifted to the right when compared with bulk FeSe_x_Te_1−x_ (00 l) reflections. Moreover, a slight shift in FeSe_x_Te_1−x_ (001) reflections to the right is observed with decreasing growth temperatures. Figure 1(e) shows the in-plane *θ–*2*θ* scan of FeSe_x_Te_1−x_ (101) reflection along with CaF_2_ (111) reflection. The (101) reflection of the FeSe_x_Te_1−x_ thin films is also shifted to the right compared to FeSe_x_Te_1−x_ bulk (101) reflection. A slight peak shift to the right is also observed for decreased growth temperatures. The peak shifts of both out-of-plane and in-plane to the right indicate that the lattice parameters of our FeSe_x_Te_1−x_ thin films are totally reduced compared to those of bulk samples.

**Figure 2:**
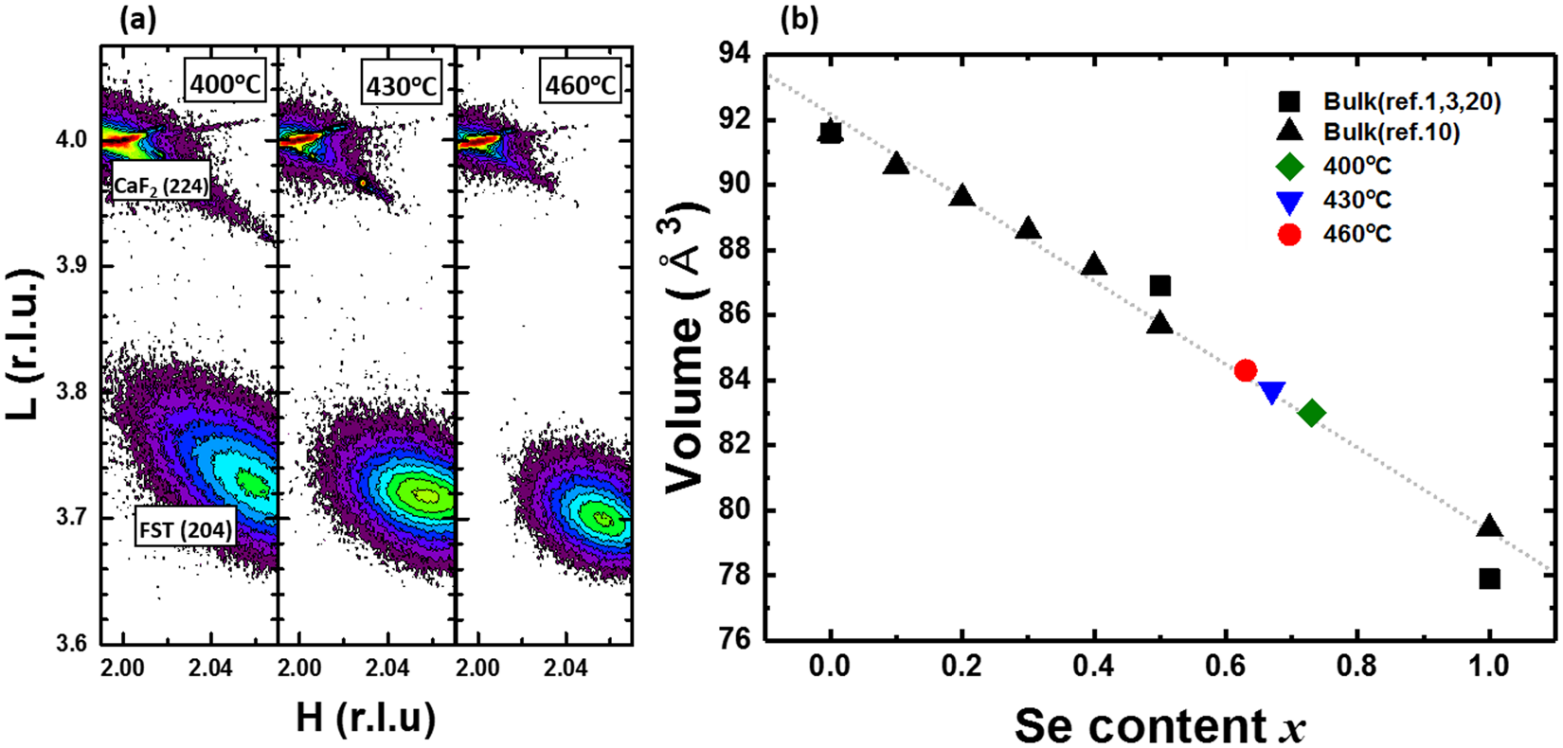
RSM analysis and unit-cell volume of FeSe_x_Te_1−x_ thin films. (**a**) Maps showing X-ray diffraction intensities around (224) reflections of CaF_2_ and (204) reflection of FeSe_x_Te_1−x_ thin films grown at 400, 430, and 460 °C, respectively. With increasing growth temperature, (204) reflections are shifted to lower right. This means that *a* and *c* lattice constants gradually increase as the growth temperature increases, (**b**) Unit-cell volumes of FeSe_x_Te_1−x_ were calculated using lattice constants of bulk FeSe_x_Te_1−x_ (solid) from a previous report and lattice constants of thin films (open) were obtained from the RSM data.

To obtain precise lattice constants of FeSe_x_Te_1−x_ films, an RSM analysis was performed. Figure 2(a) shows RSM patterns around CaF_2_ (224) reflections for FeSe_x_Te_1−x_ thin films grown at 400, 430, and 460 °C. The calculated *a* and *c* lattice constant of FeSe_x_Te_1−x_ films vary with the growth temperature as: 3.748 Å and 5.867 Å for 400 °C, 3.750 Å and 5.882 Å for 430 °C, 3.755 Å and 5.907 Å for 460 °C. The lattice constants of FeSe_x_Te_1−x_ thin films are smaller than those of the bulk target which has lattice constants of *a = *3.803 Å and *c = *6.034 Å. Furthermore, Fig. 2(a) shows that the FeSe_x_Te_1−x_ thin films and CaF_2_ substrate have different in-plane positions, indicating that the FeSe_x_Te_1−x_ thin films have no strain effect from the lattice mismatch between the film and substrate. Based on the determined lattice constants, we calculated unit-cell volumes of FeSe_x_Te_1−x_ thin films. Figure 2(b) shows the dependence of the unit cell-volume of FeSe_x_Te_1−x_ on Se content *x*. The volumes of bulk FeSe, FeTe, and FeSe_x_Te_1−x_ (0.1 ≤ x ≤ 0.5) were calculated using previously reported lattice constants^1, 3, 10, 20^. Calculation of the volume of Se rich bulk FeSe_x_Te_1−x_ (0.6 ≤ x ≤ 0.9) was impossible because of the phase separation between two types of tetragonal phases in their structures^10, 11^. However, volumes of Se rich FeSe_x_Te_1−x_ (x ≥ 0.6) are inferred by a linear fitting because volumes of FeSe_x_Te_1−x_ increase linearly with increasing Se content *x*, when *x* is below 0.5^9, 10^. The calculated volumes of our FeSe_x_Te_1−x_ thin films increase with the growth temperatures as follows: 83.0 Å^3^ (400 °C), 83.7 Å^3^ (430 °C), 84.3 Å^3^ (460 °C). When these are plotted against a matching bulk volume position, our FeSe_x_Te_1−x_ films are found to be located in the region of Se rich FeSe_x_Te_1−x_ (0.6 < x < 0.8) although these films were fabricated by using a Fe_0.94_Se_0.45_Te_0.55_ target [Fig. 2(b)]. These results indicate that lattice contraction along all axes can be attributed to an abnormal increase in Se content.

**Figure 3:**
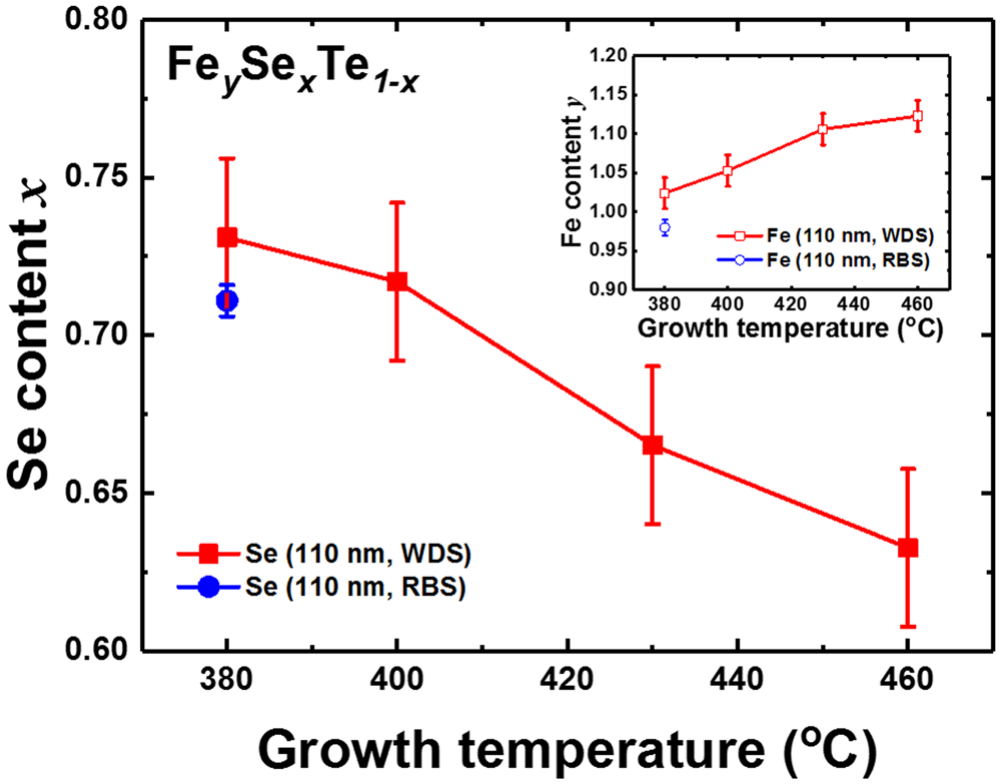
Composition analysis of FeSe_x_Te_1−x_ thin films. The WDS results of Fe_y_Se_x_Te_1−x_ thin films show the increased Se content compared to the Fe_0.94_Se_0.45_Te_0.55_ target used. With increasing growth temperature, Se content *x* decreases and Fe content *y* (inset figure) increases. Each composition measured by WDS has a standard deviation below 2% and the RBS data (circle symbol) has a standard error of ± 1% (Fe), ± 0.5% (Se) and ± 0.5% (Te).

SEM/EDS has been widely used for quantitative composition analysis. However, due to the low peak resolution of EDS hinders an accurate quantitative analysis. In fact, the limitation of accurate compositional analysis by EDS in FeSe_x_Te_1−x_ thin films on CaF_2_ because of the overlap between the energy peaks of K edge of Ca and L edge of Te has been reported^9^, which was also confirmed in our samples (see Supplementary Fig. S2). Thus, we performed WDS scan to confirm the correct composition of FeSe_x_Te_1−x_ thin films. Using WDS, the measured Se content *x* in FeSe_x_Te_1−x_ thin films are 0.731, 0.717, 0.665, and 0.633 for growth temperatures of 380, 400, 430, and 460 °C, respectively [Fig. 3]. These results indicate the significantly increased Se content *x* in FeSe_x_Te_1−x_ thin films as compared to the target composition and a slight loss of Se depending on the growth temperature. However, in the FeSe_x_Te_1−x_ thin films deposited below 400 °C, the Se content fluctuated between 0.7 and 0.73, unlike the conventional tendency of samples deposited at 400 °C (see Supplementary Fig. S3). To verify the WDS results, the composition of a FeSe_x_Te_1−x_ thin film grown at 380 °C was measured by RBS (see Supplementary Fig. S4), and the composition measured by RBS, Fe_0.98_Se_0.71_Te_0.29_ agrees well with that measured by WDS, within the error margin. Additionally, the composition of the Fe_0.94_Se_0.45_Te_0.55_ bulk target measured by WDS, was found to be Fe_0.97_Se_0.39_Te_0.61_. The difference between the measured and nominal composition of the target might be due to the high volatility of Se at high temperatures used during the fabrication of the target. Thus, the measured composition of FeSe_x_Te_1−x_ thin films shows a striking increase in the Se content, and this result agrees well with the observation of reduced volumes and lattice contraction. To the best of our knowledge, this is the first report on the observation of abnormal composition changes in FeSe_x_Te_1−x_ thin films grown by PLD.

Significantly, the main phenomenon in the abnormal composition change in FeSe_x_Te_1−x_ thin film is the change in the chalcogen ratio (Se:Te) [Fig. S5(a)], and not the loss of chalcogens [Fig. S5(b)] (see Supplementary Fig. S5). Loss of volatile components is commonly speculated during the growth of thin films on heated substrates under high vacuum, as depicted in Fig. S5(b). However, because not only the chalcogen loss is relatively small compared to the remarkable increase in Se content *x* but also the overall stoichiometry of our FeSe_x_Te_1−x_ thin films was almost maintained, the composition change is attributed to the formation of a plume when the target is irradiated with the excimer laser. In general, the plume transfers the target substances stoichiometrically to the substrate, in the PLD system. However, when a mixed target containing various substances is used, a distinction between energetic species of plume can occur, owing to the differences in the formation energy of each substance. For instance, when the Sn(Se, Te) target mixed as SnSe and SnTe = 1:1 was ablated by an excimer laser, SnTe fully dissociated and ionized during laser ablation, whereas SnSe partially vaporized congruently with ionization during ablation, because SnSe has a relatively low formation energy^21, 22^. This implies that SnSe which has a relatively stronger bond is capable of getting transferred through the plume from the target to the substrate, without dissociation. Hence, a change in composition during film growth in PLD system is most probable in a mixed material in which the components have different formation energies.

Interestingly, FeSe_x_Te_1−x_ can also be regarded as a mixed structure comprised of five types of structures and bonds, such as FeSe, FeTe, and FeSe_0.5_Te_0.5_ (see Supplementary Fig. S6), and each type of structure has a different thermodynamic property. In particular, Se rich FeSe_x_Te_1−x_ has shown phase separation between FeSe and FeSe_x_Te_1−x_ phases^10, 11^. This suggests that the separated phase is more thermodynamically stable than the single phase of Se rich FeSe_x_Te_1−x_. To compare the formation energy correctly, we have calculated the formation energies of PbO-type FeSe (α-FeSe), α-FeSe_0.5_Te_0.5_, and α-FeTe. The formation energies calculated by first principle are −3.392, −3.179, and −3.020 eV for α-FeSe, α-FeSe_0.5_Te_0.5_, and α-FeTe, respectively (see Supplementary S8 and, Tables S1 and S2). Since α-FeSe has relatively low formation energy, α-FeSe has stronger binding than α-FeTe or α-FeSe_0.5_Te_0.5_, indicating that Fe has a higher tendency to combine with Se than with Te. This indicates the high possibility of increasing Fe to Se ratio in the FeSe_x_Te_1−x_ thin films because FeSe binding is relatively more stable in the plume when the FeSe_x_Te_1−x_ target is irradiated by laser.

Although FeSe binding is relatively stable, both a slight decrease in Se content *x* and a slight increase in Fe content have been observed with increasing growth temperatures in FeSe_x_Te_1−x_ thin films [Fig. 3]. The high volatility of Se is one possible reason. When a material containing a high volatile component is deposited by PLD under the high vacuum condition, a loss of the volatile substance is possible^23, 24^. Thus, Se loss is expected because Se has lower melting point and higher vapour pressure than Te and Fe^15^. Additionally, we assume that the driving force for the phase transition of FeSe is one other reason for Se loss. FeSe undergoes a phase transition from PbO-type tetragonal structure to NiAs hexagonal structure (β) close to 450 °C in bulk system^16^. Interestingly, our growth temperature is close to 450 °C, where a phase transition is observed. When we calculated the formation energy of β-FeSe, we obtained a value higher than that of α-FeSe_x_Te_1−x_. This means β-FeSe has a relatively weaker bonding than α-FeSe_x_Te_1−x_. Thus, we presume that the Fe-Se bonding may be relatively unstable when the FeSe_x_Te_1−x_ thin film is deposited on a heated CaF_2_ substrate when the growth temperature approaches 450 °C. However, since the formation energy is calculated assuming a state of vacuum and 0 K, there is a limitation in representing the formation energy at a given temperature.

**Figure 4:**
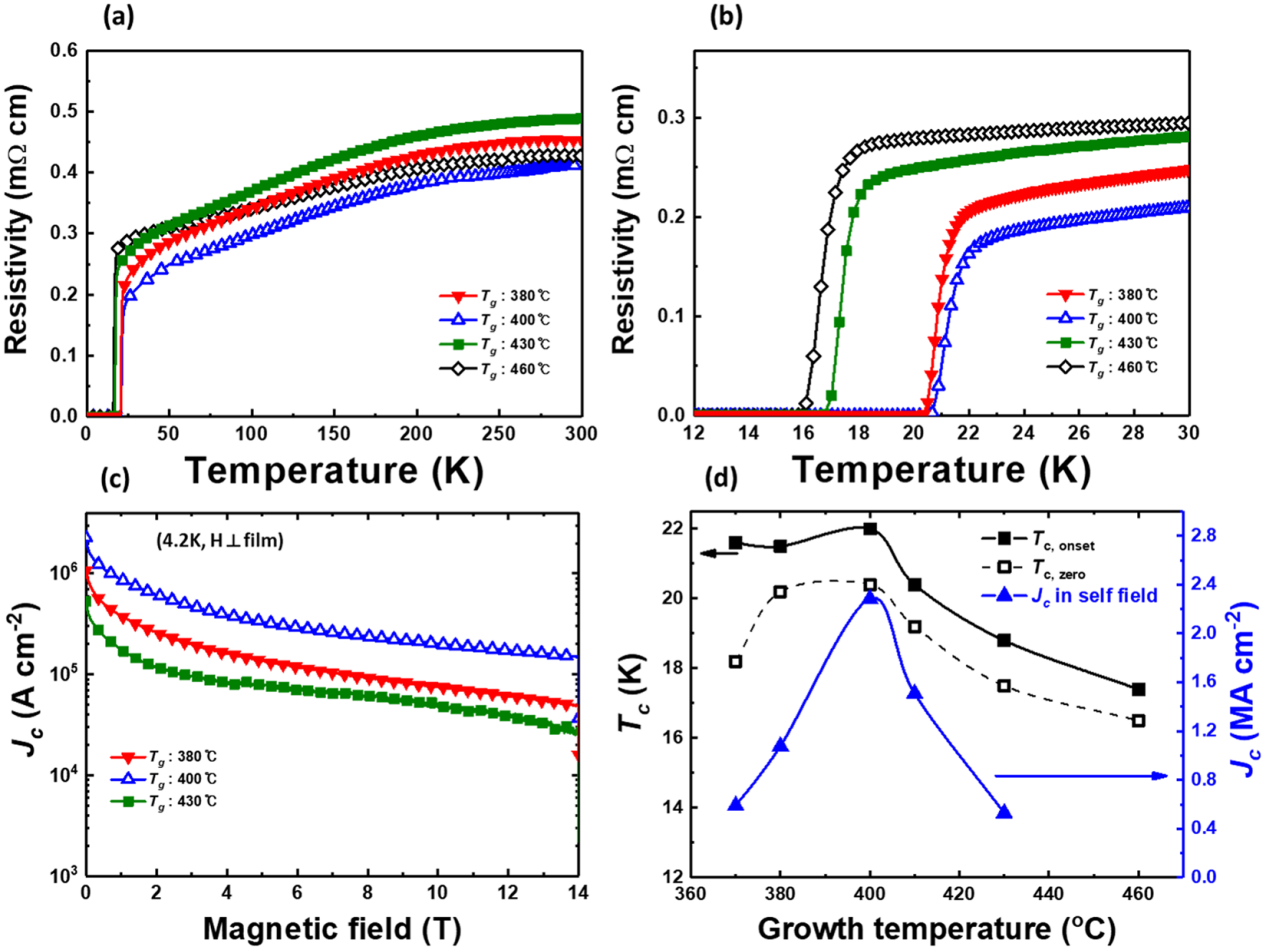
Superconducting properties of FeSe_x_Te_1−x_ thin films for different growth temperatures (*T*_g_)﻿. Temperature dependence of (**a**) the resistivity from room temperature to below *T*_c_ and (**b**) the superconducting transition of FeSe_x_Te_1−x_ thin films, (**c**) Magnetic field dependence of magnetization *J*_c_ in FeSe_x_Te_1−x_ thin films at 4.2 K, and (**d**) *J*_c_ and *T*_c_ as a function of growth temperatures.

To verify whether *T*_c_ of our FeSe_x_Te_1−x_ thin films is indeed improved by the increase in Se content *x*, the temperature dependent resistivity (*ρ*(T)) was measured using a four-point probe method. Figure 4(a,b) show the dependence of the resistivity of FeSe_x_Te_1−x_ thin films on the growth temperature. Our Se rich FeSe_x_Te_1−x_ thin films (0.6 ≤ x ≤ 0.8) have reasonable *T*_c_ as compared to those reported in literature^4–6, 9, 10^. The maximum *T*_c,onset_ and zero resistance (*T*_c,zero_) are 22.0 K and 20.4 K, respectively, for the Fe_1.05_Se_0.72_Te_0.28_ films grown at 400 °C. At growth temperatures higher than 400 °C, *T*_c,onset_ value decreases: 18.8 K (430 °C) and 17.4 K (460 °C) [Fig. 4(b)]. When FeSe_x_Te_1−x_ films were fabricated at ≤ 400 °C, the *T*_c,onset_ of FeSe_x_Te_1−x_ remained over 20 K, whereas *T*_c,zero_ randomly deteriorated to 18.2, 20.2, and 20.4 K (see Supplementary S7, Fig. S7). Interestingly, *T*_c_ increases with increasing Se content *x* in spite of the degrading crystalline quality, according to FWHM [Fig. 1(b)]. This means that the chalcogen ratio has a more significant effect on the superconducting properties than crystalline quality. Even though *T*_c_ of the FeSe_x_Te_1−x_ films is more strongly associated with chalcogen ratio than the crystalline quality, the latter too affects *T*_c,zero_ and superconducting transition. When the *g*rowth temperature is decreased below 400 °C, the crystalline quality of FeSe_x_Te_1−x_ thin films deteriorated, whereas Se content *x* of the FeSe_x_Te_1−x_ films remains almost 0.7, within the error margin [Fig. 1(b) and Supplementary Fig. S3]. These FeSe_x_Te_1−x_ films show similar *T*_c,onset_ values of over ~20 K. However, when the crystalline quality is poor, the deterioration of *T*_c,zero_ with *T*_c_ tailing is observed [Fig. S7]. Thus, it is important to fabricate highly crystalline films with the remaining Se content *x* of over 0.7 in FeSe_x_Te_1−x_ thin films. Additionally, since Fe content increased with increasing growth temperature, the effect of excess Fe is considered. Generally, excess Fe degrades *T*_c_ of FeSe_x_Te_1−x_, and this effect of excess Fe is confirmed using temperature dependence of resistivity or specific heat^18^. If the excess Fe degrades the *T*_c_ of FeSe_x_Te_1−x_, the resistivity increases as the temperature decreases to the point where the superconducting transition occurs. However, as shown in Fig. 4(a), the resistivity decreases with decreasing temperature, showing metallic behaviour. Thus, even if the content of Fe increases with increasing growth temperature, the effect of excess Fe on the superconductivity of FeSe_x_Te_1−x_ thin films is negligible because temperature dependence of resistivity of our FeSe_x_Te_1−x_ thin films shows a tendency when there is no excess Fe.

Figure 4(c) shows magnetization *J*_c_ as a function of the magnetic field for all films measured by a vibrating sample magnetometer in fields up to 14 T. FeSe_x_Te_1−x_ thin film grown at 400 °C shows a reliable *J*_c_ of 2.23 MA/cm^2^ in self-field when compared to the magnetization of FeSe_x_Te_1−x_ thin films on yttria-stabilized zirconia substrate^19^. Figure 4(d) shows the relationship between *T*_c_ and *J*_c_ as a function of the growth temperature and *J*_c_ shows a characteristic similar to *T*_c, zero_. This implies that *J*_c_ is also influenced by chalcogen ratio along with the crystalline quality of FeSe_x_Te_1−x_ thin films.

The enhancement of *T*_c_ in Se rich FeSe_x_Te_1−x_ thin films is closely related to the structural factors such as anion height, Ch-Fe-Ch bond angle (where Ch = chalcogen), and the suppression of phase separation^9, 10, 25, 26^. Among various factors, we first focused on anion height because the anion height of FeSe_x_Te_1−x_ gradually approaches 1.38 Å as the Se content *x* increases^9, 25^. As the Se content *x* increases from 0.1 to 0.5, *T*_c_ of FeSe_x_Te_1−x_ increases. When Se content *x* is increased from 0.5 to 0.9, the *T*_c_ of FeSe_x_Te_1−x_ is decreased in bulk due to the phase separation. However, *T*_c_ of Se rich FeSe_x_Te_1−x_ was enhanced when phase separation was prevented by the fabrication of thin films by PLD^9, 10^. Since our FeSe_x_Te_1−x_ thin films with enhanced *T*_c_ show Se rich FeSe_x_Te_1−x_ composition and the samples are highly epitaxial without phase separation, the enhanced *T*_c_ can also be explained by the anion height and suppression of phase separation. Secondly, we focused on bond angle of Fe and Chalcogen^18^. Bellingeri *et al*. insisted that reduced in-plane lattice contraction cause the reduced bond angle which approach to ideal value as 109.47° with enhancement of *T*_c_^4^. Since the our Se rich FeSe_x_Te_1−x_ thin films show the reduced in-plane lattice constant similar to reported lattice constant of FeSe_0.5_Te_0.5_, the increased Se content *x* sufficiently enhances the *T*_c_ of FeSe_x_Te_1−x_ thin films. As a results, the increase of the Se content *x* is a significantly important factor for improving the *T*_c_ in the FeSe_x_Te_1−x_ thin film because the anion height and bond angle become closer to the ideal values as the ratio of Se increases.

**Figure 5:**
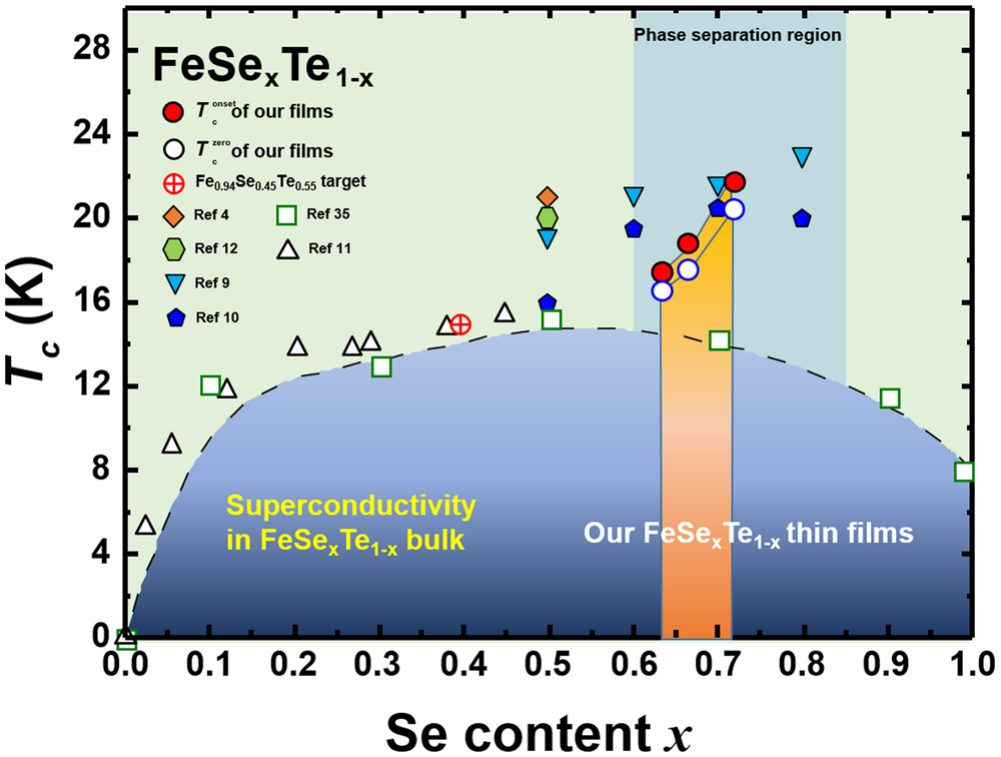
Phase diagram of *T*_c_ as a function of Se content, *x*. The open squares^35^ and up-triangles^11^ represent *T*_c_ of bulk samples from previously reported data. Circles indicate *T*_c,onset_ (solid) and *T*_c,zero_ (open) of FeSe_x_Te_1−x_ films fabricated from a Fe_0.94_Se_0.45_Te_0.55_ target (circle with cross). The FeSe_x_Te_1−x_ sample grown at 380 °C was excluded from the phase diagram due to selenium ratio fluctuations (see Supplementary Fig. S3). *T*_c_ of the Fe_0.94_Se_0.45_Te_0.55_ target (14.6 K) is plotted on the phase diagram based on the measured composition (Fe_0.97_Se_0.39_Te_0.61_). Other solid symbols show reported *T*_c_ values of FeSe_x_Te_1−x_ thin films from published reports in which the compositions were assumed to be same as the nominal target compositions^4, 9–12^.

Based on the measured composition of FeSe_x_Te_1−x_ thin films, we present a phase diagram for FeSe_x_Te_1−x_ as a function of Se content *x* [Fig. 5]. As shown in Fig. 5, *T*_c_ of the reported bulk FeSe_x_Te_1−x_ shows a dome-like tendency depending on the chalcogen ratio, and the reported *T*_c_ of FeSe_0.5_Te_0.5_ films is significantly improved over the bulk *T*_c_. However, since the compositions of the reported FeSe_0.5_Te_0.5_ thin films were based on the bulk target composition, the actual composition of these thin films may be different depending on each experimental condition. In addition, when data from our FeSe_x_Te_1−x1−x_ thin films with the actual measured composition were plotted on the phase diagram, we confirm that *T*_c_ of FeSe_x_Te_1−x_ thin films increases significantly as Se content *x* increases, contrary to that observed in FeSe_x_Te_1−x_ bulk. In general, *T*_c_ of FeSe_x_Te_1−x_ generally deteriorated when Se content *x* is over 0.8, and diverse origins have been speculated, including phase transition^9, 26–29^. However, we believe that it is possible to obtain a more elevated *T*_c_ for FeSe_x_Te_1−x_ when *x* is increased to more than 0.8 because FeSe has shown high *T*_c_ in monolayers^30^ and also under high pressures^31^.

## Conclusion

We have demonstrated that the remarkable increase in Se content *x* in FeSe_x_Te_1−x_ thin films is one of the most critical parameters for enhancing the superconductivity of FeSe_x_Te_1−x_ thin films fabricated by PLD. Although our FeSe_x_Te_1−x_ thin films were fabricated using a Fe_0.94_Se_0.45_Te_0.55_ target, the composition of the FeSe_x_Te_1−x_ thin films were not equivalent to that of the target. A Se rich FeSe_x_Te_1−x_ (0.6 < x < 0.8) composition was confirmed by the accurate WDS and RBS analyses. Although our FeSe_x_Te_1−x_ thin films have a Se rich FeSe_x_Te_1−x_ phase, which generally shows phase separation, our thin films were found to consist of a single phase. The abnormal change in the chalcogen ratio (Se:Te) is due to the preference of Fe to bond with Se because of the low formation energy. In addition, a slight decrease in Se content *x* with increasing growth temperatures was observed in FeSe_x_Te_1−x_ thin films, although the loss in Se is relatively small compared to the gain in the chalcogen ratio. However, there may be other unidentified factors affecting the composition change, because the mechanism of thin film growth in a PLD system is complicated and to the best of our knowledge, there have been no experimental reports on the abnormal change in the chalcogen ratio. Further research is required to completely understand the underlying causes of this change and hence to measure the correct compositions of the FeSe_x_Te_1−x_ thin films through more accurate measurements. We believe that these results provide the most satisfactory resolution to the controversial issues concerning the optimized chalcogen ratio and the mechanism of *T*_c_ enhancement in FeSe_x_ *T*e_1−x_ thin films. Furthermore, changes in chalcogen ratios in thin films should be an important consideration in the growth and study of various complex chalcogenide compounds.

## Materials and Method

FeSe_x_Te_1−x_ thin films were fabricated on (001)-oriented CaF_2_ substrate at different growth temperatures, which is the temperature applied to the substrate, ranging from 380 to 460 °C by PLD with KrF (248 nm) excimer laser (Coherent, COMPEX PRO 205 F) in vacuum with a base pressure of 2 × 10^−5^ Pa. The energy density of the focused laser beam, the repetition, and distance between the target and substrate are 3 J/cm^2^, 3 Hz and 4 cm, respectively. Fe_0.94_Se_0.45_Te_0.55_ targets used were prepared by an induction melting method. For structural analysis, we used a four-circle XRD (PANalytical, X’Pert pro), 2D detector XRD system (Bruker, D8 Discover with a Vantec 2D detector), and six-circle XRD for RSM (Bruker, D8 ADVANCE) using Cu-K_α1_ radiation (*λ = *1.5406 Å), and XRD in acceleration (Pohang accelerator laboratory, 3 A beamline, *λ = *1.148 Å). The composition of the films was measured by WDS (CAMECA SX51 located in UW-Madison) and calculated as an average of at least 15 scattered points on each sample. The RBS data was obtained by the Accelerator Techniques Group in EAG Laboratories and the used He^++^ ion beam energy and normal detector angle are 2.275 MeV and 160°, respectively. Formation energies were calculated *via* density functional theory (DFT) calculations as implemented in the Vienna *ab initio* simulation package (VASP) 5.2.2 code^32–34^. The normal state and critical temperature resistivity measurements were carried out in a cryostat cooled by a closed cycle refrigerator using a four-point probe method. *T*_c,onset_ describes the temperature where the resistivity reaches 90% of the normal resistivity above transition. To measure magnetization *J*_*c*_, ﻿we used a 14 T oxford vibrating sample magnetometer (VSM) by applying the magnetic field perpendicular to the surface of the films at 4.2 K. Through Bean’s model, the magnetic moment was converted to *J*_c_ according to the equation, *J*_*c*_ = 15∆m/(V_*_r), where, ∆m, V, and r are the magnetic moment, volume, and radius.

## Electronic supplementary material

supplementary information
