# Supplementary material for: Origin of the emergence of higher Tc than bulk in iron chalcogenide thin films
Source: Sci Rep. 2017 Aug 30;7:9994. doi: 10.1038/s41598-017-10383-1 (PMC5577040; doi:10.1038/s41598-017-10383-1)
Supplement: Supplementary file 1 — supplementary information [file 41598_2017_10383_MOESM1_ESM.pdf]

Supplementary information

Origin of the emergence of higher T_c than bulk in iron chalcogenide thin films

Sehun Seo[a], Jong-Hoon Kang[b], Myeong Jun Oh[c], Il-Seok Jeong[a], Jianyi Jiang[d], Genda Gu[e], Jung-Woo Lee[b], Jongmin Lee[a], Heesung Noh[a], Mengchao Liu[f], Peng Gao[f], Eric E. Hellstrom[d], Joo-Hyoung Lee[a], Youn Jung Jo[c], Chang-Beom Eom[b], and Sanghan Lee[a,1]

[a]*School of Materials Science and Engineering, Gwangju Institute of Science and Technology, Gwangju 61005, South Korea*

[b]*Department of Materials Science and Engineering, University of Wisconsin-Madison, Madison, Wisconsin 53706, USA*

[c]*Department of Physics, Kyungpook National University, Daegu, 41566, South Korea*

[d]*Applied Superconductivity Center, National High Magnetic Field Laboratory, Florida State University, Tallahassee, Florida 32310, USA*

[e]*Condensed Matter Physics and Materials Science Department, Brookhaven National Laboratory, Upton, New York 11973, USA*

[f]*Electron Microscopy Laboratory, School of Physics, Peking University, Beijing 100871, China*

Correspondence and requests for materials should be addressed to S. L. (sanghan@gist.ac.kr)

S1. Additional structural analysis for $FeSe_xTe_{1-x}$ thin films

In order to investigate the crystalline quality and epitaxial arrangement of $FeSe_xTe_{1-x}$ thin films in detail, we performed an out-of-plane θ–2θ scan of $FeSe_xTe_{1-x}$ thin films using not only four-circle X-ray diffractometer with a two dimensional (2D) area detector but also hard XRD measurement at the 3A beamline in Pohang Light Source with a six-circle PSI diffractometer.

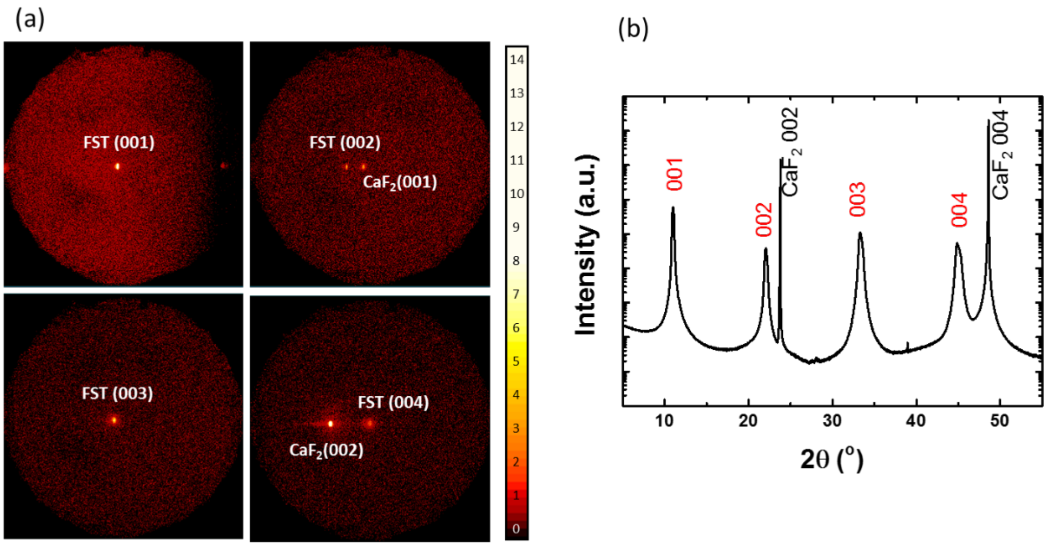

Figure S1 | Out-of-plane θ–2θ scan of $FeSe_xTe_{1-x}$ thin films by (a) 2D detector XRD and (b) XRD in the acceleration laboratory. Only film and substrate peaks are observed, indicating that our thin films are grown epitaxially, without phase separation or any secondary phases.

S2. Compositional analysis for $FeSe_xTe_{1-x}$ thin film by SEM/EDS

Scanning electron microscopy with energy dispersive spectroscopy (SEM/EDS) is widely used for the compositional analysis of thin films. When we measured the composition of $FeSe_xTe_{1-x}$ thin films grown at 380 °C on CaF_2, $SrTiO_3$ and $LaAlO_3$ substrates through EDS analysis, the measured composition of $FeSe_xTe_{1-x}$ thin films on CaF_2 and $SrTiO_3$ substrate were incorrect due to peak overlap. However, there is no energy peak overlap in $FeSe_xTe_{1-x}$ thin films on $LaAlO_3$ and the measured composition by EDS is $Fe_{0.96}Se_{0.74}Te_{0.26}$. When we verified the composition of this sample by WDS, the measured composition was found to be $Fe_{0.97}Se_{0.73}Te_{0.27}$, agreeing well with the EDS results.

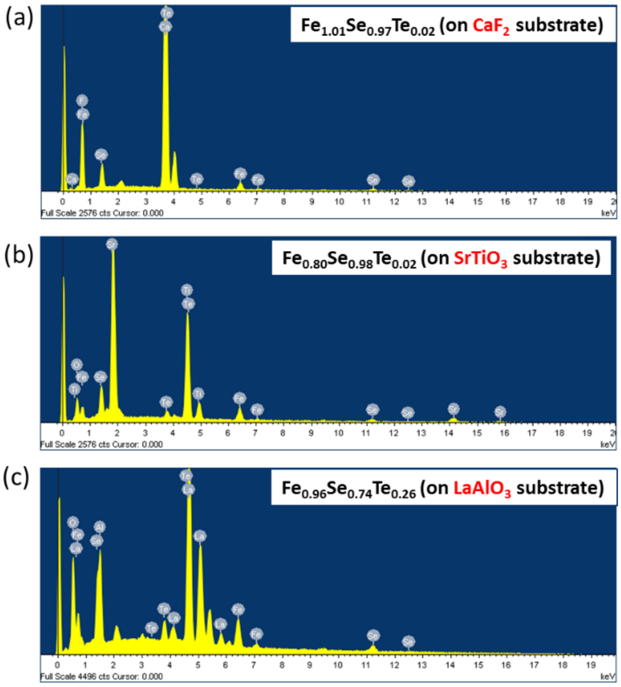

Figure S2 | SEM/EDS of $FeSe_xTe_{1-x}$ thin films grown at 400 °C (a) on CaF_2, (b) $SrTiO_3$, and (c) $LaAlO_3$ substrates.

S3. Se atomic percentage in $FeSe_xTe_{1-x}$ thin films

When we measured the composition of $FeSe_xTe_{1-x}$ thin films grown at temperatures higher than 400 °C, $FeSe_xTe_{1-x}$ thin films were found to have almost the same composition for repeated measurements. However, $FeSe_xTe_{1-x}$ thin films deposited at temperatures below 400 °C showed fluctuations in composition.

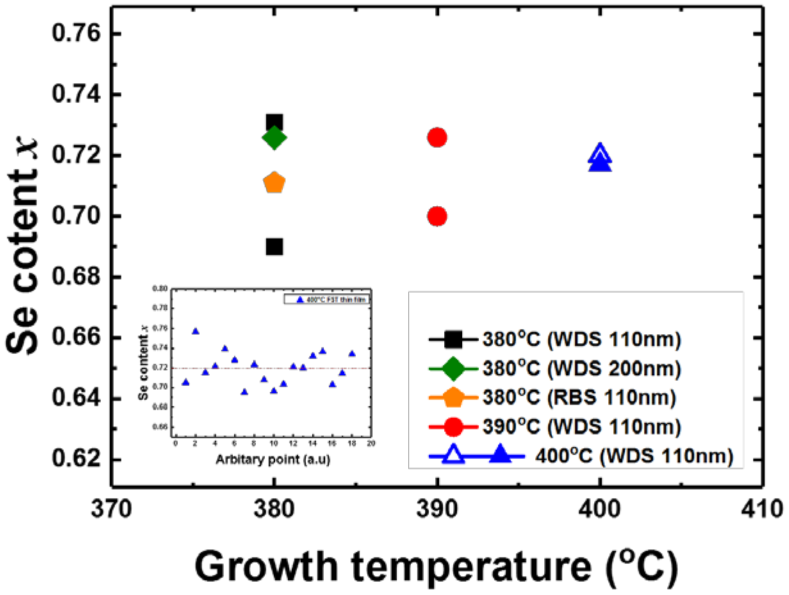

Figure S3 | WDS results of $FeSe_xTe_{1-x}$ thin films grown at temperatures lower than 400 °C on CaF_2 substrate. Inset of the figure indicates the composition of each random point on the $FeSe_xTe_{1-x}$ thin film grown at 400 °C (closed triangle). The red dotted line in the inset figure indicates the average composition of $FeSe_xTe_{1-x}$ thin films grown at 400 °C.

S4. Compositional analysis for $FeSe_xTe_{1-x}$ thin film by RBS

Rutherford backscattering spectrometry (RBS) is one of the powerful tools for measuring the precise composition of materials. When we measured the composition of $FeSe_xTe_{1-x}$ thin films grown 380 °C, the measured composition is found to be $Fe_{0.98}Se_{0.71}Te_{0.29}$. Since the theoretical modelling line corresponds with the experimental data, this result is deemed reliable.

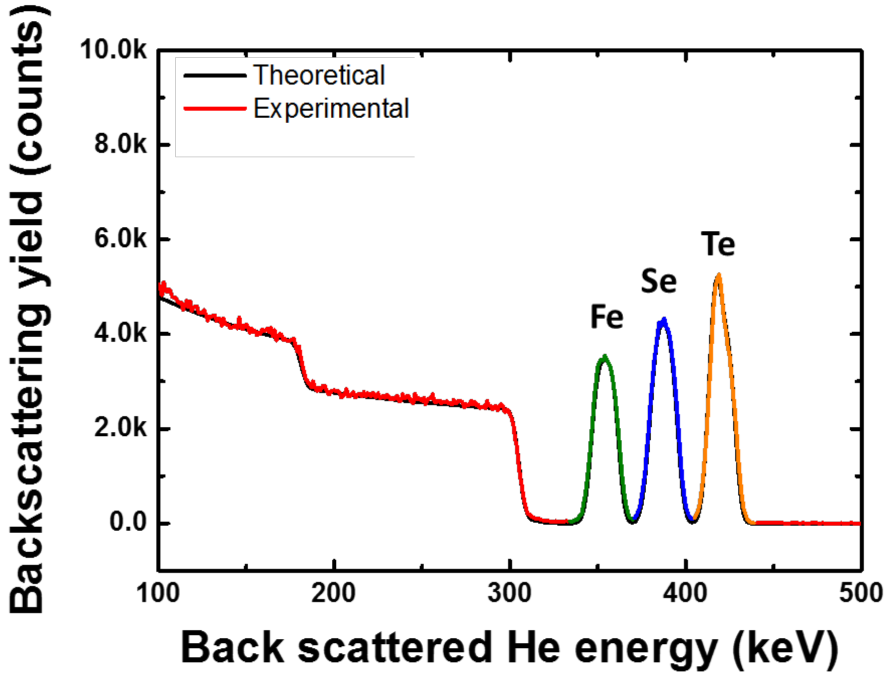

Figure S4 | RBS spectrum of a $FeSe_xTe_{1-x}$ thin film (110 nm) grown at 380 °C on CaF_2 (001) substrate. Black line indicates the theoretical spectrum for the same structure, which matches well with the experimental spectrum (coloured line).

S5. Schematic diagram of the compositional change during the thin film growth by PLD

There are two phenomena in the compositional change in $FeSe_xTe_{1-x}$ thin films. One is the loss of the volatile component. This means that the position of Se existed as a vacancy due to the volatility of Se, and hence Fe atomic ratio could increase. The other is the change in the chalcogen ratio. This means that the chalcogen ratio (Se:Te) is changed while as the ratio Fe:Ch (Ch = Chalcogen) remains 1:1.

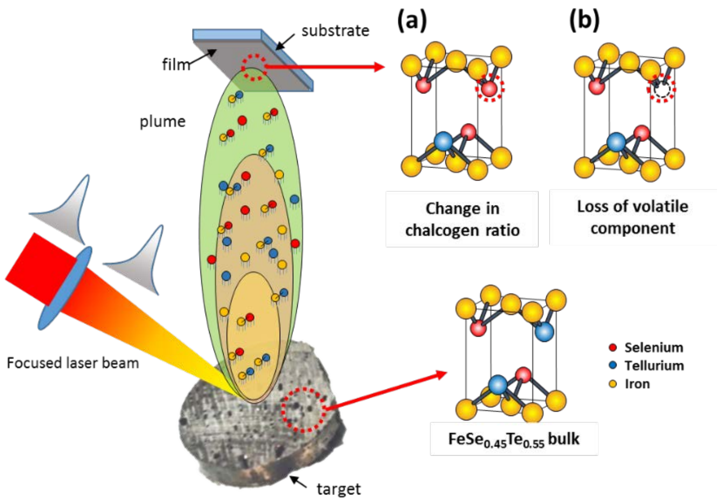

Figure S5 | Schematic diagram of the compositional change during the $FeSe_xTe_{1-x}$ thin film growth by PLD (a) Change in the chalcogen ratio. (b) Loss of the volatile component.

S6. Schematic of the $FeSe_xTe_{1-x}$ structure.

$FeSe_xTe_{1-x}$ has five types of structures such as FeSe, $FeSe_{0.25}Te_{0.75}$, $FeSe_{0.5}Te_{0.5}$, $FeSe_{0.75}Te_{0.25}$, and FeTe with a tetrahedral geometry.

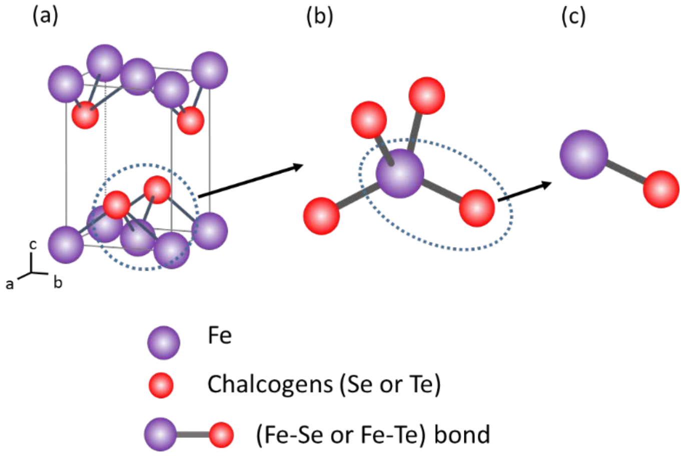

Figure S6 | (a) PbO-type tetragonal structure of ternary $FeSe_xTe_{1-x}$. The five possible structures of $FeSe_xTe_{1-x}$ based on one unit-cell are FeSe, FeTe, $FeSe_{0.5}Te_{0.5}$, $FeSe_{0.75}Te_{0.25}$ and $FeSe_{0.25}Te_{0.75}$. (b) Tetrahedral Fe_2Ch_2 (Ch = chalcogen). This tetrahedral Fe_2Ch_2 is stacked into ternary $FeSe_xTe_{1-x}$. (c) In this ternary $FeSe_xTe_{1-x}$ system, there is only Fe-Ch bond without any Fe-Fe or Ch-Ch bonds.

S7. Critical transition temperature (T_c) of $FeSe_xTe_{1-x}$ thin films

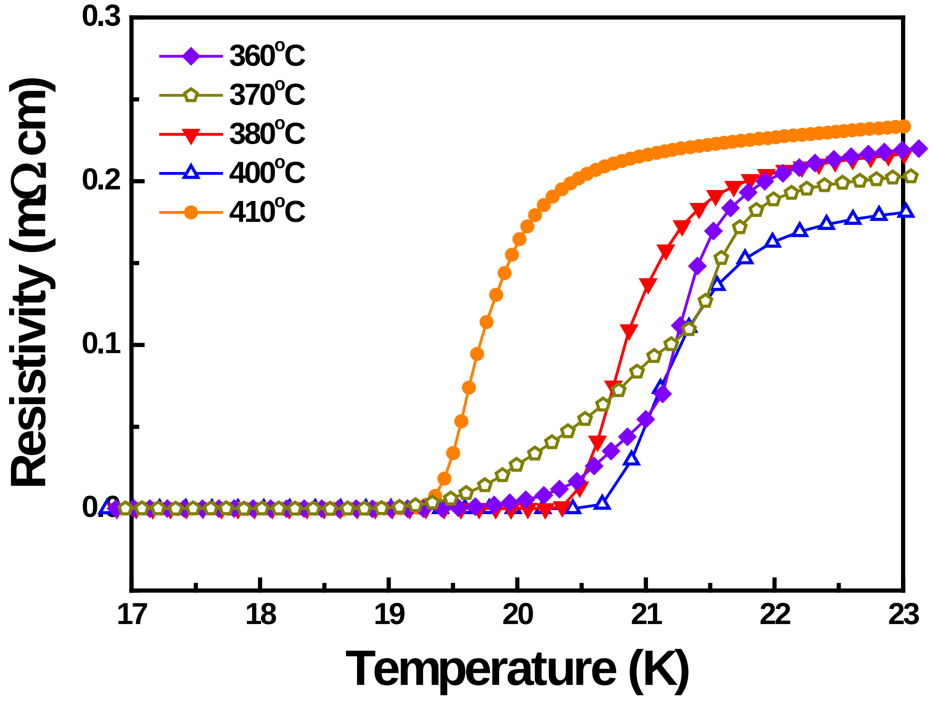

Figure S7 | T_c of 110 nm $FeSe_xTe_{1-x}$ thin films grown at a temperature less than 410 °C on CaF_2 (001) substrate. In $FeSe_xTe_{1-x}$ thin films grown at these temperatures, a tail of resistivity transition is observed.

S8. Formation energy of $FeSe_xTe_{1-x}$.

Calculations of the structural properties and formation energies of FeCh (Ch = chalcogen, Se or Te) were performed *via* density functional theory (DFT) as implemented in the Vienna *ab initio* simulation package (VASP) 5.2.2 code[1-3]. The projector augmented wave (PAW) method and the generalized gradient approximation (GGA) proposed by Perdew, Burke, and Ernzerhof [4] are adopted for the exchange-correlation function. The cut-off energies for a plane wave basis set and charge density are 800 eV and 6400 eV, respectively. The spin-polarized calculations and spin-orbit coupling calculations are included for the geometry optimization and formation energies, respectively. A mesh of 18 × 18 × 9 k-points grid centred at the Γ point was sampled for the Brillouin-zone integration. We employed the experimental lattice parameters and relaxed the atom coordinates through the conjugate-gradient method until the Hellmann-Feynmann forces acting on each atom is less than 0.001 eV/A. To consider magnetic ordering states, we adopted $\sqrt{2}a \times \sqrt{2}a \times c$, 2a × a × c, and a × a × c unit cells to calculate the collinear antiferromagnetic (AFM), bicollinear AFM, and other magnetic order states, respectively. Additionally, the 2a × 2a × c unit cell is used for α-$FeSe_{0.5}Te_{0.5}$ (PbO-type). We also considered α-FeSe, α-FeTe as the antiferromagnetic states[5]. Furthermore, we found that the ground state of α-$FeSe_{0.5}Te_{0.5}$ is a bicollinear-order AFM state after total energy calculations. In our calculations, the formation energies are classified as follows:

$$E_F(FeCh) = E_{FeCh} - E_{Fe} - E_{Ch}$$

TABLE S1. The experimental lattice constants of FeCh.

	a [Å]	c [Å]
α-FeSe [6]	3.765	5.518
β-FeSe [7]	3.617	5.883
α-FeTe [8]	3.822	6.270
α-$FeSe_{0.5}Te_{0.5}$ [9]	3.776	5.931

TABLE S2. Calculated formation energies of FeCh.

	E_{FeCh}	E_{Fe}	E_{Ch}	E_F(FeCh)
α-FeSe	-12.511	-5.638	-.3481	-3.392
β-FeSe	-11.990	-5.638	-3.481	-.2871
α-FeTe	-11.800	-5.638	-.3142	-3.020
α-$FeSe_{0.5}Te_{0.5}$	-12.129	-5.638	-3.312	-3.179

Reference

1 Kresse, G. & Hafner, J. Ab initio molecular dynamics for liquid metals. *Phys. Rev. B* **47**, 558 (1993).

2. Kresse, G. & Hafner, J. Ab initio molecular-dynamics simulation of the liquid-metal–amorphous-semiconductor transition in germanium. *Phys. Rev. B* **49,** 14251 (1994).

3. Kresse, G. & Furthmüller, J. Efficient iterative schemes for ab initio total-energy calculations using a plane-wave basis set. *Phys. Rev. B* **54,** 11169 (1996).

4. Lebegue, S. & O. Eriksson. Electronic structure of two-dimensional crystals from ab initio theory. *Phys. Rev. B* **79,** 115409 (2009)

5. Ma, F. *et al.* First-principles calculations of the electronic structure of tetragonal α-FeTe and α-FeSe crystals: Evidence for a bicollinear AFM order. *Phys. Rev. Lett.* **102**, 177003 (2009).

6. Hsu, F. C. *et al.* Superconductivity in the PbO-type structure α-FeSe. *P. Natl. Acad. Sci.* USA **105**, 14262–14264 (2008).

7. G. Song, H. Matsui, H. Akai, & H. Tabata. Structural and magnetic properties of NiAs-type FeSe and related alloy layers. *J. Vac. Sci. Technol. B* **31,** 042801 (2013)

8. Subedi, A., Zhang, L. J., Singh, D. J. & Du, M. H. Density functional study of FeS, FeSe, and FeTe: Electronic structure, magnetism, phonons, and superconductivity. *Phys. Rev. B* **78,** 134514 (2008).

9. M. K. Wu. *et al.* The development of the superconducting PbO-type β-FeSe and related compounds. *Physica C* **459**, 340 (2009)
